# An Overlooked Crisis: The Impact of COVID-19 on UK Medical Students and Their Mental Health

**DOI:** 10.1192/bjo.2022.245

**Published:** 2022-06-20

**Authors:** Aapti Shetty, Gurleen Bhatia

**Affiliations:** 1 University College London, London, United Kingdom; 2 The Tavistock and Portman NHS Foundation Trust, London, United Kingdom

## Abstract

**Aims:**

Medicine is an undoubtedly challenging degree but studying medicine during the COVID-19 pandemic has posed added challenges for medical students across the UK. With teaching being moved online, practical exams cancelled, and final year students being fast-tracked onto the NHS frontline, there has been a dramatic change in how traditional medicine is being taught- with a ‘hands-on’ approach being swapped for video calls and remote teaching. This study will highlight the impact of the COVID-19 pandemic on the mental health of medical students, how they have coped through what has been an unprecedented two years and what can be done to support them through their medical training.

**Methods:**

A cross-sectional survey was performed on medical students at University College London. This involved a combination of face-to-face interviews and an online survey. They were asked about the impact on their overall mental well-being, as well as what the most challenging aspect of studying medicine during the pandemic was. They were also asked how optimistic they feel about their future in the medical profession. The data gathered were then analysed.

**Results:**

There were 30 responses, which were a combination of face to face and an online survey. Students unanimously agreed that the most challenging aspects were loneliness, lack of face-to-face teaching and minimal social interaction. 60% stated that their mental health has suffered significantly, and everyone felt that they have missed out on certain aspects of teaching during the pandemic, namely cadaveric dissections, time on wards and gaining vital communication skills. However, the benefits of online teaching included learning at their own pace and being able to take breaks to avoid burnout.

**Conclusion:**

There are limited studies looking at the long-term effect of COVID-19 on medical students in the UK. This survey highlights the detrimental impact of the pandemic on medical training and the mental health of these students. To address the possibility of burnout before they start their medical career, more resources could be signposted by medical schools to students during this challenging time. As we are transitioning out of the pandemic, we should be mindful not to forget the cohort of students who studied medicine alone in their homes. Most importantly, we must ensure this generation of doctors is well supported as they begin to care for members of the public.

